# Inositol Inclusion Affects Growth, Body Composition, Antioxidant Performance, and Lipid Metabolism of Largemouth Bass (*Micropterus salmoides*)

**DOI:** 10.1155/2024/9944159

**Published:** 2024-01-19

**Authors:** Yinglin Xu, Ye Gong, Songlin Li, Yue Zhou, Zhixiao Ma, Ganfeng Yi, Naisong Chen, Weilong Wang, Xuxiong Huang

**Affiliations:** ^1^Key Laboratory of Agriculture Ministry for Freshwater Aquatic Genetic Research, Shanghai Ocean University, Shanghai 201306, China; ^2^China-ASEAN “The Belt and Road” Joint Laboratory of Mariculture Technology, Ministry of Science and Technology of China, Shanghai Ocean University, Shanghai 201306, China; ^3^National Demonstration Center on Experiment Teaching of Fisheries Science, Shanghai Ocean University, Shanghai 201306, China; ^4^Fantastic Victory (Shenzhen) Technological Innovation Group Co. Ltd, Shenzhen 518054, China

## Abstract

The present study explored the effects of inositol on growth performance, body composition, antioxidant performance, and lipid metabolism of largemouth bass (*Micropterus salmoides*). Six isonitrogenous and isolipidic diets containing 0 mg/kg (G1, control), 125 mg/kg (G2), 250 mg/kg (G3), 375 mg/kg (G4), 500 mg/kg (G5), and 625 mg/kg (G6) inositol were prepared and fed to cultured fish (initial weight: 110 ± 1 g) for 8 weeks in recirculating the aquaculture systems. The results indicated that compared with G1 group, the weight gain rate (WGR), specific growth rate (SGR), and feed efficiency rate (FER) in the G3 group were significantly higher. The crude lipid content of the whole fish and the liver of cultured fish was significantly reduced with increasing dietary inositol inclusion. However, no significant effects on moisture, crude protein, and ash contents of fish were observed among the different groups. Dietary inositol supplementation significantly increased muscular crude protein. However, muscular total lipid contents were decreased when the inclusion level was higher than 250 mg/kg (G3–G6 groups). As dietary inositol supplemental level increased, serum triglyceride (TG), and cholesterol (TC) contents showed an increasing trend and reached the maximum value in the G3 group. Additionally, serum low-density lipoprotein cholesterol (LDL-C) in G2, G3, G4, and G5 groups was significantly upregulated by increasing inositol. While, there was no significant change in serum high-density lipoprotein cholesterol (HDL-C) among the treatments. Inositol inclusion also significantly reduced the serum alkaline phosphatase (AKP), glutamic–pyruvic transaminase (ALT), and glutamic–oxaloacetic transaminase (AST) activities as well as serum malondialdehyde (MDA) content but significantly increased serum catalase (CAT), superoxide dismutase (SOD) activities, and total antioxidant capacity (T-AOC). Compared with the control group, the activities of hepatic total lipase (TL) and lipoprotein lipase (LPL) were significantly elevated in the G3, G4, and G5 groups. Above all, dietary inositol supplementation could improve growth performance and antioxidant capacity, and reduce the liver fat content of largemouth bass, and the optimal supplementation level of inositol in feed is estimated to be 250.31–267.27 mg/kg.

## 1. Introduction

Inositol, also known as cyclohexanol (C_6_H_12_O_6_), is an isomer of glucose and is the only bioactive cyclohexanol isomer with the most forms existing in nature [1]. Research has proved that inositol is involved in the formation of phosphatidyl inositol in living organisms, which acts as a component of cell membranes, participates in material transport, and maintains phospholipid balance [2–4]. In addition, phosphatidylinositol and its phosphorylated product phosphatidylinositol polyphosphate are precursors of the second messenger and participate in the regulation of cell physiological activities, such as DNA synthesis in lymphocytes, glycogenolysis in liver cells, amylase secretion, and insulin release, which are very important for maintaining the regular structure and physiological function of cells [5, 6].

In most aquatic animals, inositol is considered as an essential vitamin nutrient. Research has proved that an appropriate amount of inositol supplemented with aquatic feed could enhance feed efficiency, accelerate growth, and improve fat metabolism in an animal's liver and other tissues [7]. According to the present research, some fish species could synthesize inositol themselves, which could meet the growth requirement without exogenous inositol supplementation, such as channel catfish (*Ictalurus punctatus*) [8], sunshine bass (*Morone chrysops*♀ × *Morone saxatilis*♂) [9] and Atlantic salmon (*Salmo salar*) [10]. However, for those fish species, which could not synthesize inositol, such as pompano (*Trachinotus ovatus*) [11], red sea bream (*Chrysophrys major*) [12], gibel carp (*Carassius auratus gibelio*) [13], and parrot fish (*Oplegnathus fasciatus*) [14], insufficient exogenous inositol supplementation might cause slow growth, anorexia, skin ulceration, skin color darkness, stomach swelling, anemia, and other adverse symptoms. Additionally, considerable research has proved that dietary inositol inclusion could be beneficial to the antioxidant capacity of aquatic animals [15–18]. Consequently, the effect of inositol on aquatic animals varies among different species, which requires further exploration.

Largemouth bass (*Micropterus salmoides*), which originated in the United States, has become a popular freshwater aquaculture species since introduced to China in 1894 and the production could reach 800,000 tons in 2022 [19, 20]. Currently, the application and effects of inositol on largemouth bass have not been reported. Consequently, the present study was conducted to explore the impact of dietary inositol inclusion on largemouth bass, aiming to determine the inositol requirement of largemouth bass and provide a scientific basis for efficient feed production for largemouth bass.

## 2. Materials and Methods

### 2.1. Experiment Diets

The basal diet was designed as shown in Table 1. On this basis, six isonitrogenous and isolipidic diets containing 0 mg/kg (G1, control group), 125 mg/kg (G2), 250 mg/kg (G3), 375 mg/kg (G4), 500 mg/kg (G5), and 625 mg/kg (G6) inositol (purity of 99%) supplementation, respectively, were prepared. All ingredients were ground and sieved through an 80-mesh screen. The bulk ingredients and premixes were precisely weighed according to the formulated ratios and meticulously blended to follow sequential steps. The fats were then added and thoroughly mixed in a blending machine. After that, a control of 20%–30% water was added to the diet and thoroughly mixed to make a stiff dough, which was further cut into particles measuring 3–5 mm. Extruded pellets were cooked in a drying oven at 105°C for 15 min, then dried in a ventilated oven at 55°C. Then, the experiment diets were stored at −20°C until being used.

### 2.2. Experimental Procedure

All the experiment fish were obtained from Shanghai Nonghao Feed Co., Ltd. and were temporarily reared for 2 weeks feeding the basal experiment diet. After the domestication, 360 healthy largemouth bass juveniles (110 ± 1 g, disinfected with 3% salt water) were randomly allocated to 18 tanks in recirculating aquaculture systems with 20 fish per tank. During the feeding trial, each treatment fish was fed with an experimental diet twice daily until the fish were apparent satiation for 8 weeks. The water temperature was between 24 and 29°C, the ammonia nitrogen concentration was below 0.15 mg/L, the pH was 7.0–7.4, and the dissolved oxygen was above 7.0 mg/L.

### 2.3. Sample Collection

Before sample collection, the fish were starved for 24 hr for further sampling. The survival numbers of the fish were counted for survival rate (SR) calculation. The total body weight of the fish was measured for relative calculations. Then, 10 fish were randomly selected from each barrel and anesthetized with eugenol (1 : 10,000). Three of the chosen fish were taken for body composition analysis, and the remaining seven were taken on the ice tray for body length and weight measurement. Then, the tail venous blood of the seven fish was taken, and the serum was separated by centrifugation after resting on ice (4,000 *r*/min, 10 min, 4°C), which was then transferred to a 2 mL centrifuge tube with a pipette and stored at −80°C for the determination of serum biochemical indexes. After that, the liver and visceral of the seven fish were stripped and weighted for hepatosomatic index (HSI) and viscerosomatic index (VSI) calculation. Then, the sampled liver and back muscles were taken and stored at −80°C for muscle composition and liver biochemical indexes analysis.

### 2.4. Biochemical Analysis

The composition of fish was analyzed following the description by Wang et al. [21]. In brief, moisture was determined by the 105°C constant weight method (GB/T6435-2006), crude ash was determined by Marfut furnace burning (550°C; GB/T6438-1992), crude protein was determined by Kjeldahl nitrogen determination method (GB/T 6432-2018), and total lipid was determined by the chloroform–methanol extraction method.

The serum and liver biochemical indices were analyzed following the methods of Li et al. [22] and Chen et al. [23], including the activities of catalase (CAT), superoxide dismutase (SOD), glutamic–pyruvic transaminase (ALT), alkaline phosphatase (AKP), glutamic–oxaloacetic transaminase (AST), fatty acid synthase (FAS), lipoprotein lipase (LPL), hepatic lipase (HL), and total lipase (TL); and the content oftotal antioxidant capacity (T-AOC), malondialdehyde (MDA), high-density lipoprotein cholesterol (HDL-C), triglyceride (TG), cholesterol (TC), and low-density lipoprotein cholesterol (LDL-C) were analyzed using the commercial kits (Nanjing Jiancheng Bioengineering Institute) following the instructions.

### 2.5. Calculation and Statistical Analysis

The relevant variables were obtained using the formula described by Han et al. [24], and the details are listed under Table 2.

All statistical assessments were performed using SPSS 22.0. All the data were analyzed by one-way analysis of variance (ANOVA) and Duncan's multiple comparison test. *P* < 0.05 was considered as the data of different groups had significant differences. The optimum supplemental level of inositol in the feed was evaluated by regression analysis of the broken line model.

## 3. Results

### 3.1. Effects of Different Inositol Levels on Growth Performance, Feed Coefficient, and Morphological Indices of Largemouth Bass

**Figure 1:**
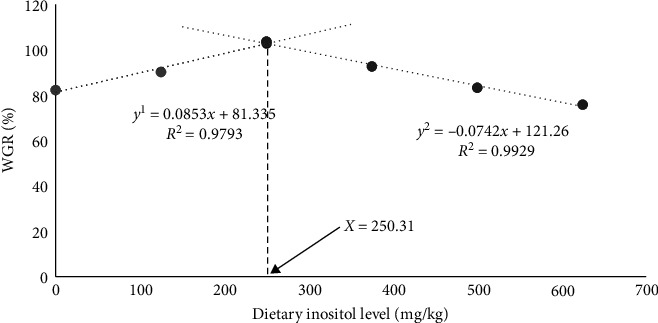
Effect of inositol on WGR of largemouth bass (*Micropterus salmoides*).

**Figure 2:**
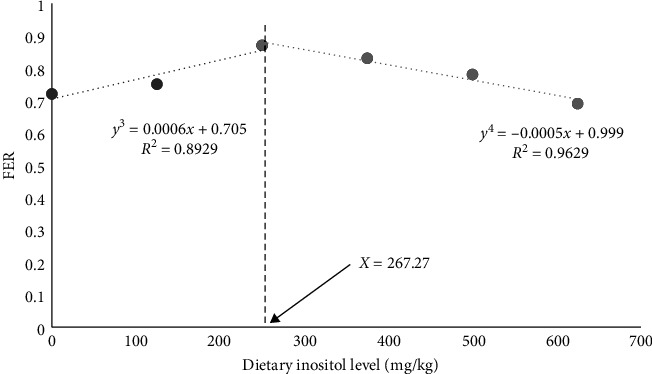
Effect of inositol on FER of largemouth bass (*Micropterus salmoides*).

The results showed that inositol inclusion had no significant effects on SR and conditional factor (CF) among different treatment groups (*P* > 0.05; Table 2). The final body weight (FBW), weight gain rate (WGR), specific growth rate (SGR), and feed efficiency rate (FER) were significantly higher in the G3 group than in the G1 group (*P* < 0.05; Table 2). Additionally, inositol supplementation significantly reduced the HSI of cultured fish (*P* < 0.05; Table 2). As to VSI, the G3–G6 groups were significantly lower than the control group (*P* < 0.05; Table 2). The results of regression model analysis between the WGR, FER, and dietary inositol levels showed that the diet with an inositol content of 250.31 mg/kg had the best WGR for largemouth bass (Figure 1), and the diet with an inositol content of 267.27 mg/kg had the highest FER for largemouth bass (Figure 2). These results indicated that based on the growth-promoting effects, the best dietary inositol supplemental level was 250.31–267.27 mg/kg.

### 3.2. Effects of Different Inositol Levels in Diet on the Composition of Whole Body, Liver, and Muscle of Largemouth Bass

The total lipid contents of whole fish, muscle, and liver of largemouth bass in G3–G6 group were significantly decreased compared with the control group (*P* < 0.05; Table 3). Additionally, inositol inclusion significantly increased the crude protein level in the muscle of largemouth bass (*P* < 0.05; Table 3). No obvious changes in the moisture and ash of the whole fish and muscle were observed among different groups (*P* > 0.05; Table 3).

### 3.3. Effects of Different Dietary Inositol Levels on Serum Biochemical Indices of Largemouth Bass

The results showed that G3 group had a significantly higher TG, TC, and LDL-C contents (*P* < 0.05; Table 4). However, no obvious difference in HDL-C levels among different dietary groups were observed (*P* > 0.05; Table 4). Additionally, the activities of AKP and AST significantly decreased with dietary inositol inclusion (*P* < 0.05; Table 4). The ALT activity significantly decreased in the G3–G5 groups compared with G1 group (*P* < 0.05; Table 4).

### 3.4. Effects of Different Dietary Inositol Levels on the Antioxidant Capacity of Largemouth Bass

Dietary inositol supplementation significantly decreases the serum MDA content of cultured fish (*P* < 0.05; Table 5). Dietary inositol inclusion in G2–G5 group could also observably increase the activities of serum SOD, CAT, and the T-AOC compared with the control group (*P* < 0.05; Table 5).

### 3.5. Effects of Different Dietary Inositol Levels on Liver Lipid Metabolism of Largemouth Bass

The activities of LPL and TL were significantly higher in G3–G5 groups than the G1 group (*P* < 0.05; Table 6). In addition, there was no significant difference in the activities of HL and FAS (*P* > 0.05; Table 6), but the HL activity showed an upward trend (Table 6).

## 4. Discussion

Inositol is usually used as a nutritional additive in aquatic feed, which could promote the growth of fish and improve the feed efficiency. The results of the present study showed that there was no significant influence on the SR and no obvious inositol deficiency of cultured largemouth bass feeding with different inositol supplementation levels, which was consistent with the previous studies [8–10]. However, whether largemouth bass could synthesize inositol to satisfy the requirement still need further research. Additionally, the WGR, SGR, and FER of largemouth bass feedin a diet supplemented with 250 mg/kg inositol increased significantly. These results were consistent with the study on golden pompano (*Trachinotus ovatus*) [15], juvenile barramundi (*Lates calcarifer* Bloch) [25], juvenile tilapia (*Oreochromis niloticus × Oreochromis aureus*) [26], juvenile Chinese mitten crab (*Eriocheir sinensis*) [27], and Juvenile hybird grouper (Brown-marbled Grouper *Epinephelus fuscoguttatus ♀* × giant grouper *E. lanceolatus* ♂) [28]. Studies have pointed out that inositol is involved in the synthesis of phosphatidyl inositol, and phosphatidyl inositol could generate inositol triphosphate through phosphorylation, which could regulate the concentration of calcium ions and control the release of acetylcholine. Acetylcholine could make intestinal contraction frequent through neural pathways, accelerate gastrointestinal emptying, shorten the satiation time, and increase the food intake of fish, thus increasing the WGR [29]. Inositol is also a cyclohexane derivative and has a strong affinity with lipids, which could promote lipid metabolism, and improve the utilization of feed lipid, thus improving feed efficiency and accelerating growth. In the present study, from the viewpoint of WGR and FER, largemouth bass displayed the best growth performance when the diets added 250.32 and 267.27 mg/kg inositol, respectively. However, when the dietary inositol inclusion level reached 625 mg/kg, the WGR of cultured largemouth bass was lower than the control group. Above all, the dietary inositol requirement is closely dependent on fish species, growth stages, and feed formulation [30–33].

Besides the growth performance, dietary inositol inclusion also affected the body composition of cultured fish. In this study, inositol supplementation significantly reduced the fish's lipid content. However, the research on gibel carp (*C. auratus gibelio*) [13], parrot fish (*O. fasciatus*) [14], and Pacific white shrimp (*Litopenaeus vannamei*) [34] showed that inositol could increase the body lipid content. Additionally, the study on Atlantic salmon (*S. Salar*) [10], grass carp (*Ctenopharyngodon idella*) [35], and Siberian taimen (*Hucho taimen*) [36] demonstrated that dietary inositol did not change the body lipid content. Consequently, the regulation of inositol on the body's lipid metabolism varied among different fish species. Additionally, in the present study, 250–500 mg/kg dietary inositol inclusion could significantly decrease liver lipid content and increase the activity of liver LPL and TL of largemouth bass, which was consistent with the study of Cui et al. [33] and Zhang et al. [37]. Studies have demonstrated that liver lipid deposition would cause fatty liver, which has negative effects on the growth performance, feed utilization, immunity, and stress tolerance of the aquatic animals [37–39]. Consequently, inositol could effectively relieve liver fat accumulation by accelerating liver lipid catabolism.

No matter if it is the exogenous lipids or the endogenous lipids, they need to be transported through the blood to reach the whole body, so the blood lipid level could directly express the body's lipids metabolism. This study indicated that compared with the control group, 250 mg/kg inositol significantly increased serum TG content. This result was in opposition to the research on Chinese sucker (*Myxocyprinus asiaticus*) [40], which might be due to the different effects of inositol on fish species. In addition, 250 mg/kg inositol supplementation also significantly increased TC and LDL-C contents, which was in correspondence with the results of Jiang's research on Chinese sucker (*Myxocyprinus asiaticus*) [40]. This might be because inositol promoted lipolysis in the liver and produced more saturated fatty acids and increased liver TC content, which further bound to the LDL produced by the liver and transported through the blood. Thus, inositol inclusion increased serum TC and LDL-C content. Apart from serum lipid level, the activities of AKP, ALT, and AST are also essential blood biochemical indices, which could be an indicator of animal health [41]. In detail, when liver damage occurs, a rapid increase in serum ALT and AST levels could be observed [42]. The increased level of serum AKP would cause membrane permeability and integrity damage, which further cause cell damage [43]. In this study, the activities of AKP, ALT, and AST in blood decreased significantly when the inositol supplemental level was 250–500 mg/kg. This result was in line with Pan et al. [16] and Wu et al. [32]. However, when the level of inositol supplementation reached 625 mg/kg, the activities of ALT and AST in the blood increased, indicating that appropriate inositol could protect liver health and improve the liver function of largemouth bass, but excess inositol supplementation could not have the beneficial effects.

MDA, combined with SOD and CAT, are often used to detect the organism's oxidation degree, which could reflect the antioxidant capacity [44, 45]. In the present study, dietary inositol inclusion could significantly reduce the serum MDA content and significantly increased the activities of serum SOD and CAT. These results were in line with Jiang et al. [7] on *Cyprinus carpio* and Zhang et al. [36] on *H. taimen*. T-AOC serves as an indicator of antioxidant defense levels of both enzyme and nonenzyme components [34]. Results of the present showed that inositol inclusion could significantly increase the serum T-AOC of cultured largemouth bass. Above all, dietary inositol could improve the antioxidant capacity and reduce the damage caused by oxidative stress. However, when the inositol supplementation level was more than 500 mg/kg, the activities of SOD and CAT and the T-AOC showed a decreasing trend, indicating that excessive inositol supplementation would cause a decrease in antioxidant capacity.

## 5. Conclusion

Above all, dietary inositol supplementation could improve the growth performance and antioxidant capacity, regulate blood lipids, reduce liver fat content, and improve the liver function of largemouth bass. In the present study, according to the regression analysis, the optimal dosage of inositol in feed was estimated to be 250.31–267.27 mg/kg.

## Figures and Tables

**Table 1:** Basal diets formula and nutrition content (DM basis).

Items	Content (%)
Ingredients	
Fish meal	42
Wheat gluten meal	3
Blood powder	4
Shrimp meal	5
Fermented soybean meal	9
Corn gluten meal	11
Squid paste	2
Beer yeast	2
* α*-Starch	8
Soybean oil	4
Soybean lecithin	2.5
V-mix^1^	1
M-mix^2^	1
Cr_2_O_3_	0.5
Ca(H_2_PO_4_)_2_	1
Choline chloride	0.2
Zeolite	3.8
Total	100
Proximate nutrient composition	
Crude protein	50.51
Crude lipid	9.76
Ash	15.45
Moisture	4.20

Vitamin premix and mineral premix were purchased from Guangzhou Liankun Biotechnology Company and its inositol was removed. ^1^V-mix (mg/kg of diet) contained: VA 10,000 IU, VB1 30, VB2 60, VC 800, pyridoxine HCl 20, VB12 0.1, biotin 2.5, VD3 2,000 IU, VE 160 IU, menadione 40, folic acid 10, inositol 100, niacin 200, and calcium pantothenate 100. ^2^Mineral mix (g/kg of diet) contained: FeC_6_H_5_O_7_·5H_2_O 0.181, MgSO_4_·7H_2_O 1.09, KH_2_PO_4_ 0.932, NaH_2_PO_4_·2H_2_O 0.432, AlCl_3_·6H_2_O 0.051, ZnCl_2_ 0.08, CuSO_4_·5H_2_O 0.063, MnSO_4_·H_2_O 0.031, KI 0.028, CoCl_2_·6H_2_O 0.006, and NaSeO_3_·3H_2_O 0.0008.

**Table 2:** Effects of different inositol levels in diet on growth performance, feed utilization, and morphological indexes of largemouth bass (*Micropterus salmoides*).

Item	Dietary treatment					
	G1	G2	G3	G4	G5	G6
IBW (g)	110.02 ± 0.25	109.18 ± 0.13	109.97 ± 0.06	110.07 ± 0.10	109.77 ± 0.52	109.98 ± 0.08
FBW (g)	200.67 ± 5.42^bc^	208.66 ± 14.36^bc^	224.00 ± 2.97^a^	215.33 ± 7.77^ab^	207.60 ± 2.71^bc^	196.32 ± 8.45^c^
WGR (%)	82.23 ± 5.16^b^	90.23 ± 3.27^ab^	103.56 ± 2.61^a^	92.64 ± 5.96^ab^	83.22 ± 6.45^b^	75.79 ± 3.33^b^
SGR (%/d)	1.00 ± 0.05^b^	1.07 ± 0.11^ab^	1.18 ± 0.02^a^	1.09 ± 0.10^ab^	1.01 ± 0.11^b^	0.94 ± 0.08^b^
SR (%)	100 ± 0.00	100 ± 0.00	100 ± 0.00	98 ± 2.89	97 ± 5.77	98 ± 2.89
FER	0.72 ± 0.04^b^	0.75 ± 0.12^ab^	0.87 ± 0.03^a^	0.83 ± 0.07^ab^	0.78 ± 0.06^ab^	0.69 ± 0.07^b^
CF (g/cm^3^)	9.56 ± 0.59	9.88 ± 0.52	10.11 ± 0.26	10.01 ± 0.62	9.73 ± 0.62	9.2 ± 0.12
HSI (%)	1.21 ± 0.02^a^	1.10 ± 0.01^b^	1.08 ± 0.06^b^	1.07 ± 0.03^b^	1.07 ± 0.01^b^	0.95 ± 0.02^c^
VSI (%)	6.08 ± 0.14^a^	5.90 ± 0.13^ab^	5.36 ± 0.01^c^	5.51 ± 0.15^bc^	5.54 ± 0.16^bc^	5.56 ± 0.01^bc^

Duncan's multiple range test was performed in all groups, and values within a row with a common superscript letter are not significantly different from the other dietary groups (*P* > 0.05; mean ± SEM; *n* = 3). Survival rate (SR, %) = final fish number/initial fish number × 100. Feed efficiency ratio (FER) = increased body weight/dry feed consumed. Weight gain rate (WGR, %) = (final body weight − initial body weight)/initial body weight × 100. Specific growth rate (SGR, %/d) = Ln (final body weight/initial body weight)/experimental days × 100. Hepatosomatic index (HSI, %) = liver weight/body weight × 100. Viscerosomatic index (VSI, %) = viscera weight/body weight × 100. Condition factor (CF) = final body weight (g)/length (cm)^3^ × 100.

**Table 3:** Effects of dietary inositol level in diet on the composition (% wet weight) of whole body, muscle, and liver of largemouth bass (*Micropterus salmoides*).

Item	Dietary treatment					
	G1	G2	G3	G4	G5	G6
Whole body						
Moisture	70.38 ± 1.71	71.64 ± 2.36	71.44 ± 0.47	70.55 ± 0.47	71.02 ± 1.34	71.67 ± 2.54
Crude protein	17.55 ± 0.27	16.91 ± 0.35	17.02 ± 0.59	17.77 ± 0.71	17.81 ± 0.39	17.35 ± 0.48
Crude lipid	5.9 ± 0.31^a^	5.39 ± 0.11^b^	5.39 ± 0.56^b^	5.12 ± 0.35^b^	5.1 ± 0.27^b^	4.94 ± 0.24^b^
Ash	3.74 ± 0.34	3.78 ± 0.45	4.1 ± 0.29	4.03 ± 0.25	4.0 ± 0.36	3.85 ± 0.38
Muscle						
Moisture	78.23 ± 0.49	77.79 ± 0.67	77.7 ± 0.29	77.69 ± 1.36	78.05 ± 0.39	77.9 ± 0.59
Crude protein	19.08 ± 0.48^b^	19.55 ± 0.41^a^	19.6 ± 0.58^a^	19.85 ± 0.49^a^	19.54 ± 0.8^a^	19.73 ± 0.26^a^
Crude lipid	1.11 ± 0.03^a^	1.07 ± 0.09^ab^	1.04 ± 0.08^b^	1.05 ± 0.05^b^	1.03 ± 0.02^b^	1.02 ± 0.08^b^
Ash	1.32 ± 0.12	1.24 ± 0.13	1.24 ± 0.08	1.32 ± 0.08	1.3 ± 0.1	1.31 ± 0.09
Liver						
Moisture	70.13 ± 0.2	70.9 ± 0.07	70.95 ± 0.09	70.13 ± 0.11	69.79 ± 0.08	70.06 ± 0.12
Crude lipid	5.18 ± 0.11^a^	4.35 ± 0.48^b^	3.34 ± 0.15^c^	4.38 ± 0.17^b^	4.41 ± 0.15^b^	4.04 ± 0.18^b^

Duncan's multiple range test was performed in all groups, and values within a row with a common superscript letter are not significantly different from the other dietary groups (*P* > 0.05; mean ± SEM; *n* = 3).

**Table 4:** Effects of dietary inositol level in diet on serum biochemical indices of largemouth bass (*Micropterus salmoides*).

Item	Dietary treatment					
	G1	G2	G3	G4	G5	G6
TG (mmol/L)	2.47 ± 0.11^c^	2.56 ± 0.29^bc^	3.03 ± 0.13^a^	2.91 ± 0.32^ab^	2.27 ± 0.05^c^	2.21 ± 0.25^c^
TC (mmol/L)	4.7 ± 0.11^c^	4.81 ± 0.30^c^	5.57 ± 0.10^a^	4.95 ± 0.22^bc^	5.25 ± 0.22^ab^	4.81 ± 0.10^c^
HDL-C (mmol/L)	2.17 ± 0.18	2.35 ± 0.21	2.35 ± 0.36	2.14 ± 0.16	2.09 ± 0.18	2.22 ± 0.25
LDL-C (mmol/L)	1.25 ± 0.09^bc^	1.66 ± 0.31^a^	1.88 ± 0.13^a^	1.55 ± 0.13^ab^	1.8 ± 0.1^a^	1.2 ± 0.15^c^
AKP (U/L)	8.92 ± 0.96^a^	6.36 ± 0.18^b^	6.92 ± 0.09^b^	7.14 ± 0.51^b^	6.71 ± 0.65^b^	6.15 ± 0.27^b^
ALT (U/L)	6.89 ± 0.45^a^	5.58 ± 0.42^ab^	5.07 ± 0.43^b^	4.37 ± 0.79^b^	4.5 ± 0.82^b^	5.44 ± 0.49^a^
AST (U/L)	17.44 ± 0.90^a^	13.11 ± 1.86^b^	11.3 ± 1.36^b^	12.3 ± 0.97^b^	10.33 ± 1.89^b^	12.08 ± 2.03^b^

Duncan's multiple range test was performed in all groups, and values within a row with a common superscript letter are not significantly different from the other dietary groups (*P* > 0.05; mean ± SEM; *n* = 3). TG, triglyceride; TC, total cholesterol; HDL-C, high-density lipoprotein cholesterol; LDL-C, low-density lipoprotein cholesterol; AKP, alkaline phosphatase; ALT, alanine transaminase; AST, aspartate aminotransferase.

**Table 5:** Effects of dietary inositol level in diet on antioxidant capacity of largemouth bass (*Micropterus salmoides*).

Item	Dietary treatment					
	G1	G2	G3	G4	G5	G6
MDA (nmol/mL)	31.85 ± 1.57^a^	27.26 ± 0.71^b^	25.59 ± 0.91^bc^	20.15 ± 0.46^d^	26.51 ± 1.15^bc^	23.9 ± 1.31^c^
CAT (U/mL)	4.51 ± 0.56^b^	6.07 ± 0.81^a^	6.23 ± 0.41^a^	6.3 ± 1.29^a^	6.19 ± 0.74^a^	5.71 ± 0.56^ab^
SOD (U/mL)	80.11 ± 4.11^c^	99.60 ± 2.89^ab^	102.59 ± 3.33^ab^	105.47 ± 4.68^a^	97.68 ± 3.37^b^	96.49 ± 2.9^b^
T-AOC (U/mL)	1.4 ± 0.05^c^	1.67 ± 0.04^a^	1.68 ± 0.02^a^	1.68 ± 0.02^a^	1.53 ± 0.02^b^	1.54 ± 0.05^b^

Duncan's multiple range test was performed in all groups, and values within a row with a common superscript letter are not significantly different from the other dietary groups (*P* > 0.05; mean ± SEM; *n* = 3). MDA, malondialdehyde; CAT, catalase; SOD, superoxide dismutase; T-AOC, total antioxidant capacity.

**Table 6:** Effects of different inositol levels in diet on liver metabolism of largemouth bass (*Micropterus salmoides*).

Item	Dietary treatment					
	G1	G2	G3	G4	G5	G6
LPL (U/mg prot)	1.47 ± 0.16^b^	2 ± 0.15^ab^	2.66 ± 0.11^a^	2.76 ± 0.26^a^	2.82 ± 0.13^a^	2.19 ± 0.11^ab^
HL (U/mg prot)	1.69 ± 0.51	1.98 ± 0.08	1.85 ± 0.15	2.37 ± 0.03	2.18 ± 0.21	1.93 ± 0.16
TL (U/mg prot)	3.16 ± 0.21^b^	3.97 ± 0.22^ab^	4.57 ± 0.13^a^	5.13 ± 0.27^a^	4.84 ± 0.25^a^	4.11 ± 0.24^ab^
FAS (ng/mL)	338.25 ± 6.08	376.81 ± 5.97	362.38 ± 5.65	336.2 ± 5.53	323.67 ± 4.55	340.8 ± 3.53

Duncan's multiple range test was performed in all groups, and values within a row with a common superscript letter are not significantly different from the other dietary groups (*P* > 0.05; mean ± SEM; *n* = 3). LPL, lipoprotein lipase; HL, hepatic lipase; TL, total lipase; FAS, fatty acid synthetase.

## Data Availability

The data that support the findings of this study are available from the corresponding author upon reasonable request.
